# The Plague, Its Origin and Disappearance

**Published:** 1855-09

**Authors:** Aug. Theod. Stamm


### MEDICAL EXAMINER.

NEW SERIES.—NO. CXXIX.—SEPTEMBER, 1855.

### ORIGINAL COMMUNICATIONS.

### *The Plague, its Origin and Disappearance.*

By Aug. Theod. Stamm.

It is the object of this paper to give some information of the most destructive of all diseases—a disease which for centuries and centuries, resisting the efforts of the most learned physicians, has produced the greatest misery and many scenes of deepest immorality and degradation. No one has yet explained the total disappearance, for the last ten years, of this scourge ; neither has the real place of its origin ever been demonstrated. I do not pretend to know the nature of the specific plague poison ; no one has yet recognised its essence. But from my own observations in the native country of the Plague, I hope finally to prove where this poison was year after year generated, and by what means it has been eradicated.

The Plague, Λοῖμος, Pestis seu Pestis bubonaria orientalis, Typhus pestis seu Typhus pestilentialis, die Pest, la Peste, Typhus d'Orient, Peste, Pestilenza, Pestilencia, etc., was a disease chiefly at home in Syria, Asia Minor, European Turkey and Egypt, but often spreading over the whole coast of the Mediterranean, and even over the whole of Europe, penetrating far into the interior of Russia, and when once there unchecked by the northern winter of Moscow.

It is difficult to decide whether the Plague be identical with the epidemics recorded by Moses, David, Homer, Thucydides, Herodotus, Dionysius of Halicarnassus and others, or whether the beginning of this disease may be found in the Justinian Plague, which from 542–594 raged almost without interruption in all parts of the Roman Empire, because in these old writers the symptoms are not described with such accuracy as to arrive at quite certain results.

Thus much we know for certain, that since the time of the Justinian Plague the disease has repeatedly visited not only large countries, but almost the whole of the old world, and only since this time it seems to be characterized by buboes; and after once having adopted this form it appeared indestructible, raging for more than a thousand years, and in some places never disappearing.

I do not intend to enter here into a minute description of the Plague, but only mention that it is a fever of the most aggravated kind, chiefly characterized by buboes or inflammation, swelling and suppuration of the lymphatic glands, although, according to the special properties of the disease, many varying symptoms have been observed; thus at the commencement of the Plague, cases occur where nothing is to be observed but the symptoms of a severe typhus fever, the patient generally dying in the course of a day, or from apoplexy (apoplexia pestilentialis) after a few hours of great prostration, whilst towards the end of an epidemic it assumes often such a mild form that a slight swelling of the lymphatic glands and a mild fever is all that can be observed.

Those who want more accurate information of the different forms and symptoms may find it in the writings and reports of Thomas Sydenham, Heberden, Diemerbrock, Nathan Hodges, Pestalozzi, Chicoyneau, Beerwinckel, Rothman, Melzer, Patrick Russel, Mead, Chirac, Enrico di Wolmar, Verney, Deidier, Bertrand, Souliers, Clot-Bey, Bulard, Lachèze, Gaetani-Bey and numerous other medical men, many of whom not only treated and observed the disease, but in their heroism and scientific zeal exposed themselves with undaunted courage to the danger of post mortem examinations.

*The treatment of the Plague.*—The opinion of physicians in regard to the treatment of the Plague, were but too often in contradictory opposition. The antiphlogistic method had a most celebrated defender in Thomas Sydenham; but, after more accurate inquiry, it was found to be erroneous. Chicoyneau, as well as Audon, Deidier, Verney and the other physicians at Marseilles, were much opposed to this method. Russell and Enrico di Wolmar did not reject bleeding, but whether they ever derived real advantage from it, remains, at least, very doubtful. Palloni was in favor of calomel; but, according to Bulard, calomel is always thrown off without effect, by vomiting or stools. Paris thinks that nothing but cold water should be employed. Savaresi, Bulard, Pugnet had recourse to a slightly stimulating system, and tried ether, alcohol, camphor, ammonia, etc., but success did not recommend these remedies.

The system of evacuating the stomach and bowels has, if properly administered, frequently proved beneficial; emetics and strong cathartics, given at the beginning of the disease, have been considered the best remedies. The diuretics have been recommended by Sydenham to be employed in combination with the antiphlogistic treatment; and Mead is also a defender of the diuretic method. The invigorating system in which the cordials were used had for some time a great reputation, but from their want of success their use was quite abandoned. Narcotics, too, have been tried; opium, strychnia, lactucarium, and belladonna have been given. Dr. Aubert prescribed Hachisch in the Egyptian Plague of 1833. Frictions with oil and various other treatments have never done very much for the cure of the disease. In the treatment of the buboes and carbuncles, science was likewise at fault, and did not yield successful remedies; the simple chirurgical method has proved as good as any other. But, however treated, about two-thirds of those attacked have always died. Emetics and cathartics, given at the beginning, cleanliness, cold water and demulcent drinks would probably, if generally employed, have yielded the best results. The poison once generated, is mightier than all the remedies used against it, and coming like a scourge upon careless man, should teach him that the great aim should be prevention rather than the cure of disease.

*The generating of the Plague.*—Great aridity after copious rain and inundations, winds constantly blowing from the same direction, sudden change of the seasons, scarcity of food, etc., have been found concurrent with the entrance of the Plague, and the variation of those conditions seemed often connected with the course and character of the disease. But how uncertain such indications are, is manifest, if we consider that frequently in the midst of prosperity, and during the most agreeable weather, the Plague attacked populations quite unconscious of the dreadful evil so near them. Such striking contrasts annihilated all theories in reference to its generation, and, at those times, when, during shorter or longer intervals, the disease was spread over the whole of Europe, the people were lost in the most uncertain conjectures.

At last the more civilized nations of Europe inclined to the opinion that the East and Egypt were the principal countries of the Plague, as this epidemic was found to be endemic there, even at times when the more cultivated and cleaner parts of Europe were exempt; and as the intense contagious power of this disease had manifested itself most terribly, and cases of plague frequently occurred in consequence of the intercourse of men and the exchange of merchandize along the infected frontier, it was resolved that, in order to prevent its introduction, the quarantine should be established, and that if the disease should, notwithstanding, be introduced, a *cordon sanitaire* should be drawn around the infected district—measures which were executed with the approbation of the inhabitants, who were in constant dread of an outbreak of the epidemic.

After this it became evident that the plague-poison came always from the East and Egypt towards the protected frontiers, whilst formerly it wandered through Europe in all directions, longitudinally and latitudinally.

This is in itself sufficient to contradict the views of ancient and modern physicians, who wish to maintain that the disease is alone dependent upon the condition of the atmosphere and the change of weather and season.

The real sphere of action for the Plague being in this way confined to the East and Egypt, that is to say to the Turkish Empire, in which no mutual quarantine existed, the controversy sprung up where, in this Empire, the exact spot for generating the disease was to be found.

The lower part of the Danube, Constantinople, Smyrna,

Trebisond, the capitals of Barbary, Cairo, etc., were distinguished as places where the Plague was chiefly at home, and one of the cities often accused the other of being the generator of the epidemic.

Egypt, which exhibits so many peculiarities in its natural conditions, had indeed for a long time been looked upon as the native place of the Plague, but even sensible and learned writers opposed this view ; thus, Prosper Alpinus says that the Plague is imported into Egypt from Greece, Syria and Barbary, whilst Volney, Enrico di Wolmar, Olivier and others thought that the real seat of this disease was to be found at Constantinople, because there it was never quite extinguished for any length of time.

The controversy where in the Turkish Empire the Plague was generated became the more confused the more interest the inhabitants of each city and country had in clearing themselves from such a reproach. The disease indeed usually came from Alexandria to Constantinople, still several instances occurred where Alexandria was infected by Constantinople. Yet at last the opinion gained ground that, however favorable to the Plague was the filth of Constantinople and other places, it could there only be considered as an exotic, indigenous to and coming from Egypt. Thus L. Ch. Roche wrote an article in the *Dictionnaire de Médecine et de Chirurgie pratiques*, t. xii. 1834, by which he tries to prove that the plague is indigenous to the country of the Pharaohs, citing, in his favor, the opinions of Desgenettes, Larrey, Pugnet, Savaresi, (physician to the French-Egyptian army,) Pariset and Lagasquie, (members of a commission in the year 1828, sent by the French government for the study of the disease,) Paris, Fodéré, etc.

But if, according to the opinion of most physicians, we have to accept Egypt as the country whence the Plague has most frequently extended itself; the question again arises if in Egypt the cause at once originated all over the land, or if it spread from certain fixed points. In reference to this question, it becomes evident, after earnest inquiry, that during past epidemics, Cairo and the villages of the Delta, near this city, were generally attacked first and suffered most. The reason of this phenomenon may easily be found in the condition and situation of the place.

Cairo has several hundred thousand inhabitants. Before the watering and sweeping of the streets was introduced by the vice-roy Mehemed Ali, they were full of filth. A canal which goes through the city and receives all kinds of refuse was very much neglected; its borders had always been considered as most unhealthy and most frequented by the plague. Moreover, Cairo was surrounded with an almost complete circle of hills, 150 to 300 feet in height, and where these ceased, by a projection of the Mokattam mountain. Thus purifying winds were cut off from the city. The disease always appeared after the receding waters of the Nile had left much animal and vegetable matter decaying and producing miasmata under the combined influence of heat and moisture, and after raging several months, disappeared with the Nucta, (a heavy dew,) and the scorching rays of the June sun. Nowhere, throughout Egypt, was the generating of such miasmata more favored than in and around Cairo; the refuse of several hundred thousand inhabitants, the neglected canal, and a marsh near the city, exhaled their poisonous gases, which, at once, ripening in this basin surrounded by hills, seized upon thousands of people, whose neglected corpses increased the corruption of the air. In this way, first Cairo and the villages around it were desolated, then other parts of Egypt, the Turkish Empire, and before the introduction of the protective quarantine and *cordons sanitaires,* almost the whole of the old world; the disease travelling in all directions, communicating itself to healthy places. Thus the Plague of Marseilles infected other parts of the province and southern France, but it came, nevertheless, originally, from Egypt to Marseilles.

*The disappearance of the Plague.*—Mehemed Ali gave orders to clean the city, and to water and sweep the streets every morning, but the state of health did not materially improve. It had already been remarked by physicians of the army at the time of the French-Egyptian expedition, that the encircled position of the city, combined with other unfavorable circumstances, must be very unhealthy. Advisers of Mehemed Ali repeated the remark, and the vice-roy, who was a tyrant, but seldom shrinking from the extent of an enterprise, took the bold resolution of carrying down a large portion of the earthy hills into the lowest fields, which, after having sufficiently elevated, he intended to water artificially and to convert into beautiful gardens. As once the Pharaohs dragged thousands of men to the erection of temples and pyramids, so Mehemed Ali forced thousands of fellahs (Egyptian peasants) to execute his gigantic plan.

Many died under the excessive labor, but the ranks were filled by others, and the work itself was always advancing. Thus a long chain of hills was carried down and miasmatic fields converted into charming olive and fruit gardens. And as the work progressed, the health of Cairo improved. Already in 1843, but a few cases of Plague occurred; and the disease, no longer brought from Egypt to other parts of the Turkish Empire, disappeared. At the end of the year 1844, while in Egypt, I could find no plague, and only a few cases of slight swelling of the lymphatic glands. I was assured that the disease would certainly reappear in the beginning of 1845; and going to the southern parts of the country, I expected, at my return to Cairo, to find it; yet since that time there has been no return of it. All this shows, conclusively, that Egypt was the real seat of the pestilence; and that not the whole country, but this encircled spot, at the beginning of the Delta, generated this dreadful disease. Its disappearance, after the falling of the Nucta and the entrance of the summer-heat, is also easily to be understood, if we observe the natural phenomena. The falling dew purifies the air, and the excessive heat immediately following, dries up all the vapors and all putrid matter, scorching over dead bodies and rapidly converting decaying matter into a mere powder.

Yet I do not mean to say that this or a similar epidemic might not, under favorable circumstances, find its origin also in other southern countries, chiefly where war is raging, where food is scarce, where swamps abound, or swarms of dying locusts and other impurities poison the air; but I assert, man has it always in his power to prevent the disease. War and uncleanliness can be avoided, food can be provided by wise measures taken in time, swamps can be drained, the locusts can be buried in the earth, thus doubling the fertility of the soil. Mehemed Ali has proved what can be done even under the most unfavorable circumstances, by his grand and energetic measures in improving the health of one city; and by thus destroying the germ of this most destructive of all diseases, he has unconsciously saved the lives of millions.

May the extirpation of the Plague contribute to extend the application of the great principle, that the best remedy for diseases and for all other evils, is their prevention rather than their cure.

